# Adult-Onset Autoimmune Enteropathy: A Case Report

**DOI:** 10.7759/cureus.39677

**Published:** 2023-05-30

**Authors:** Ruben D Lorentsen, Lene B Riis, Casper Steenholdt

**Affiliations:** 1 Department of Gastroenterology, Herlev and Gentofte Hospital, Herlev, DNK; 2 Department of Pathology, Herlev and Gentofte Hospital, Herlev, DNK; 3 Department of Clinical Medicine, University of Copenhagen, Copenhagen, DNK

**Keywords:** malabsorption, diarrhoea, celiac disease, villous atrophy, autoimmune enteropathy

## Abstract

Small bowel villous atrophy is most often caused by celiac disease in the Western world, but other diseases should be explored in patients without positive serology. Adult-onset autoimmune enteropathy (AIE) is a rare cause of villous atrophy first known in children with T-cell dysregulation but also seen in adults with autoimmune predispositions. Here, an 82-year-old woman with autoimmune thyroiditis was admitted with weight loss and watery diarrhoea not responding to diet change. Endoscopy revealed villous atrophy both in the duodenum and in the ileum, but no positive celiac serology. A diagnosis of autoimmune enteropathy was made based on chronic diarrhoea not responding to diet change, autoimmune predisposition, villous atrophy, typical histological findings, and no evidence of immunodeficiency or medications causing villous atrophy. The patient was treated to good effect with corticosteroids but needed total parenteral nutrition while admitted. AIE should be considered in villous atrophy without positive celiac serology.

## Introduction

Autoimmune enteropathy (AIE) is a rare cause of chronic, secretory diarrhoea caused by small bowel villous atrophy. It was first recognised in infants by Walker-Smith and Unsworth as part of immunodysregulation polyendocrinopathy enteropathy X-linked (IPEX) [1]. Later recognised in adults [2], the first proposed diagnostic criteria were severe diarrhoea, small villous atrophy, no response to any dietary restrictions (especially gluten), anti-enterocyte antibodies, and/or a predisposition to autoimmunity and absence of immunodeficiency, e.g., hypogammaglobulinemia or common variable immunodeficiency disorder (CVID) [3]. Refined criteria have been proposed, implying characteristic histological findings (villous blunting, deep crypt lymphocytosis, abnormal apoptosis, and minimal intraepithelial lymphocytosis) and the absence of other causes for villous atrophy as additional major criteria for the diagnosis, while gut-specific antibodies are no longer required for the diagnosis [4,5]. Medications (olmesartan, non-steroidal anti-inflammatory drugs (NSAIDs), immune checkpoint blockade therapy, particularly CTLA-4 antibodies) have since been shown to give rise to a secondary form of AIE [6]. Although the condition is quite rare, histologic patterns seem to be heterogenous and are proposed to be subdivided into four distinct subtypes (acute chronic duodenitis, celiac disease-like, graft-vs.-host disease-like, and mixed/no pattern) [7]. The diagnosis of AIE remains a challenge as there is considerable overlap between the differential diagnoses of other diseases with villous atrophy, e.g., celiac disease (CD), refractory CD, seronegative CD, and medication-associated enteropathy [8-10]. Only a few case series have been published, and further knowledge is warranted [3,4,7]. Here we present a rare case of AIE.

## Case presentation

An 82-year-old woman was referred to a tertiary hospital for evaluation of a suspected treatment-refractory *Clostridioides difficile (C. difficile)* infection. The patient was known to have autoimmune thyroiditis, hypertension, and osteoporosis, had had curative surgery for malignant melanoma five years ago, and had not received any immune therapies. She had stopped taking losartan half a year before presentation and did not take any other medications with a known correlation to AIE.

Four weeks prior to admission, the patient presented with sudden-onset watery diarrhoea and was found positive for *C. difficile*. She was thus treated with a two-week course of vancomycin (125 mg every other day (QOD)). However, the diarrhoea persisted, and the patient was admitted with 20-25 watery bowel movements per day, abdominal pain worsening after food intake, and dehydration with a weight loss of 5%.

At the time of admission, a repeat stool PCR examination was negative for *C. difficile* and 19 other common intestinal pathogens. A false-negative *C. difficile* infection with toxic megacolon was suspected, but a CT scan showed normal organs with fluid content in the colon and no bowel dilations. Blood tests were normal apart from low albumin (3.2 g/dL), hypopotassaemia (3.0 mM), and acute renal insufficiency (creatinine 210 mM or 2.38 mg/dL), congruent with dehydration.

The patient was next evaluated by combined ileocolonoscopy and esophagogastroduodenoscopy, with normal findings in the colon but the notable flattening of villi in the duodenum and terminal ileum.

Celiac antibodies were normal (<1 kU/L tissue transglutaminase IgA, <1 kU/L deamidated gliadin peptide IgG, normal levels of IgA, IgG, and IgM).

The patient was treated with total parenteral nutrition while waiting for histological results. Despite no oral intake, she still had 10-15 bowel movements per day and watery diarrhoea. Histology was available on day 15 of admission.

**Figure 1:**
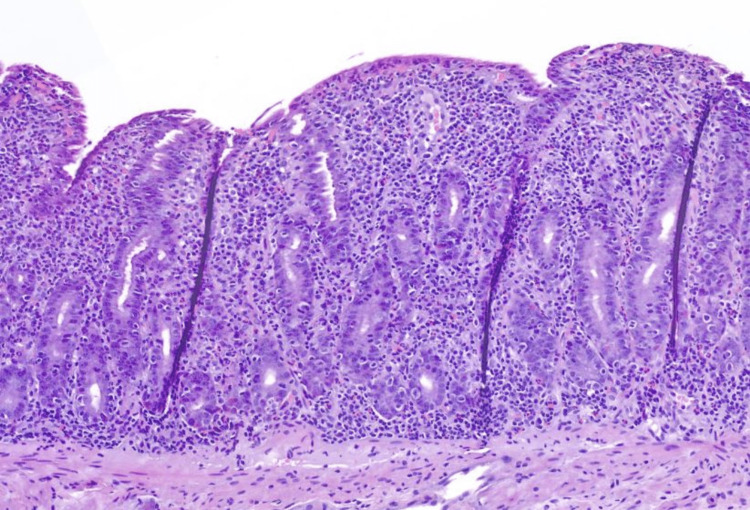
Histological examination of the duodenal biopsy with villous atrophy Celiac disease-like pattern in a duodenal biopsy with villous atrophy, increased intraepithelial lymphocytes, and diffuse inflammation in the lamina propia

Histological examination of duodenal biopsies showed blunting of the villi, crypt lymphocytosis, and intraepithelial lymphocytosis in the surface epithelium (>40 lymphocytes/100 enterocytes). In addition, there was severe chronic inflammation in the lamina propia (Figure 1).

**Figure 2:**
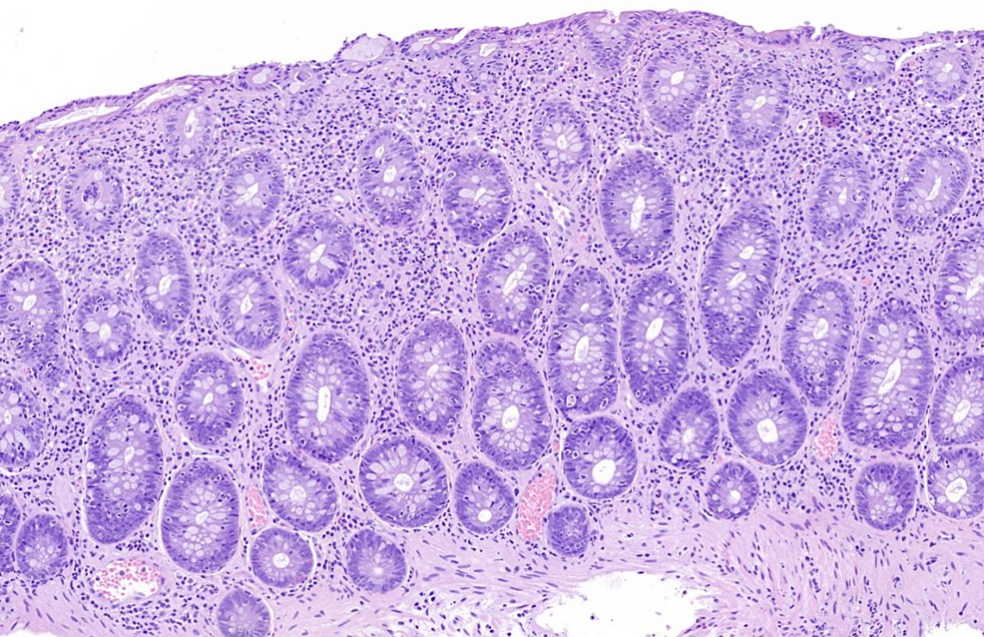
Colonic biopsy Colonic biopsy with intraepithelial lymphocytes, increased crypt epithelial apoptosis, and expansion of the lamina propia

**Figure 3:**
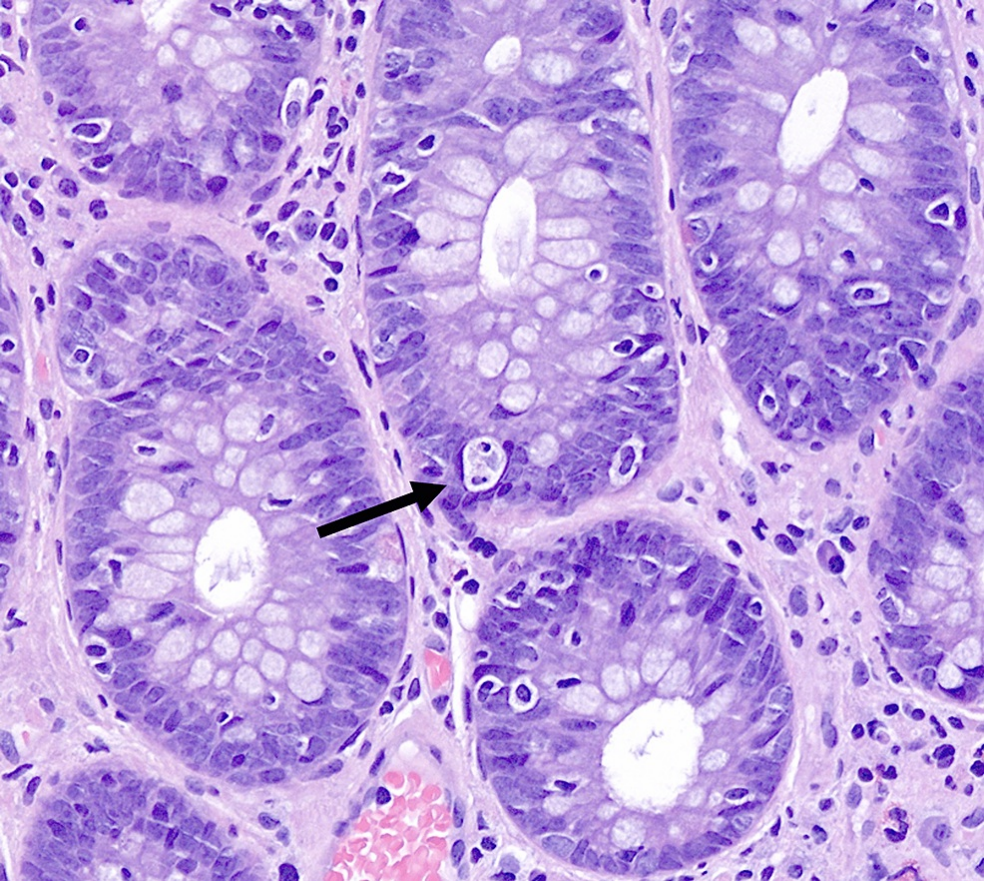
Colonic biopsy Crypt epithelial apoptosis (arrow)

The same changes were seen in biopsies from the terminal ileum, and colonic biopsies showed marked apoptosis, intraepithelial lymfocytosis, and diffuse inflammation (Figures 2-3). Anti-erythrocyte antibodies were not assessed as these are not available in Denmark.

Based on chronic secretory diarrhoea, typical histological findings congruent with celiac disease-like autoimmune enteropathy, autoimmune predisposition, no evidence of celiac disease or immunodeficiencies, and no secondary medication causes, a diagnosis of adult-onset autoimmune enteropathy was concluded, and the patient started treatment with iv methylprednisolone 60 mg per day for six days, which effectively normalised her bowel movements after two days of admission. The patient was subsequently discharged seven days after having started IV prednisolone with an oral tapering regimen consisting of oral 40 mg prednisolone tapered to 5 mg every week. After the normalisation of her bowel movements, she was quickly tapered off parenteral nutrition. However, six weeks after having tapered off prednisolone, a relapse of symptoms presented. Due to the severe side effects of prednisolone, the patient was treated with an eight-week course of 9 mg budesonide (in an open capsule to treat the whole small intestine) and initiated long-term maintenance therapy with azathioprine 50 mg and allopurinol 100 mg (normal TPMT genotype and phenotype) [4].

## Discussion

Adult AIH is a rare disease with very limited retrospective data consisting mostly of case reports and a few small case series of 13-30 patients [4,5,7]. The diagnostic criteria are still under debate, and autoantibodies are not always present, i.e., anti-enterocyte antibodies in 50%-80% of patients and anti-goblet cell antibodies in 30% [6,9,10]. The underlying pathophysiology is best known in paediatric variants (i.e., IPEX and autoimmune polyendocrinopathy, candidiasis, and ectodermal dystrophy (APECED) syndromes) with defects in T-cell regulation, while AIE in adults is mostly seen in patients with other autoimmune diseases and appears to be caused by intestinal immune dysfunction [6]. Histology of AIE was characterised by Masia et al. in a case series into four subtypes: active chronic enteritis (52%), celiac disease-like (20%), graft-versus-host disease-like (16%), and mixed/no predominant pattern (12%). They did find abnormal histopathology of the tubular gut other than the small intestine in all patients, with colonic inflammation present in 64% of patients [7].

In Western countries, celiac disease should be considered the most likely cause of small bowel villous atrophy, with 95% of patients with villous atrophy in an Italian referral centre having celiac disease [11]. Adult AIH should be considered in patients presenting with chronic, secretory diarrhoea and villous atrophy after the exclusion of celiac disease, inflammatory bowel disease, immunodeficiencies, and infectious enteritis [8,10]. The diagnosis of AIE is supported by histology, positive autoantibodies, and/or known autoimmune disease(s). Diarrhoea, malabsorption, and weight loss in AIH can be severe and even fatal, and while a diagnostic workup is done, patients may need total parenteral nutrition [3,4].

In this case of adult AIH, a *C. difficile* infection was present at admission. Triggering factors for the onset of AIH are not known, and to our knowledge, this is the second case of AIH associated with *C. difficile* infection; the other case was treated with metronidazole [12].

When the diagnosis of AIH is established, treatment is casuistic, with different immunomodulatory or immunosuppressants used, most commonly, steroids [3,4]. One-third of patients endure long-term drug-free remission. In refractory cases, autologous stem cell transplantation has been done with success [4].

## Conclusions

This case illustrates the importance of considering even rare aetiologies of chronic diarrhoea. AIH is a rare and heterogeneous disease that should be considered by clinicians when malabsorption and small intestinal villous atrophy are found in patients after the exclusion of celiac disease. The disease is rare, with risks of fatal outcomes, and treatment can be challenging. Reporting cases is warranted for better understanding.
